# Verification of the accuracy of dynamic navigation for conventional and mouthpiece methods: in vivo study

**DOI:** 10.1186/s12903-024-04327-1

**Published:** 2024-05-22

**Authors:** Koudai Nagata, Manabu Okubo, Kurumi Saito, Toshifumi Nakashizu, Mihoko Atsumi, Hiromasa Kawana

**Affiliations:** 1 Department of Oral and Maxillofacial Implantology, Kanagawa Dental University, 82 Inaoka-cho, Yokosuka, 238-8580 Japan; 2 Division of the Dental Practice Support, Kanagawa Dental University, 82 Inaoka-cho, Yokosuka, 238-8580 Japan

**Keywords:** Dental implants, Computer-aided design, Computer-aided manufacturing, Dental instruments, Fiducial markers

## Abstract

**Background:**

Dynamic navigation for implant placement is becoming popular under the concept of top-down treatment. The purpose of this study is to verify the accuracy of a dynamic navigation system for implant placement.

**Methods:**

Implant placement was performed on 38 patients using 50 implant fixtures. Patients in group C were treated using a conventional method, in which thermoplastic clips were fixed to the teeth, and patients in group M were treated using thermoplastic clips fixed to a mouthpiece attached to the teeth. The groups were compared to verify whether an accuracy difference existed. A treatment planning support program for dental implants was used to superimpose the postoperative computed tomography data on the preoperative implant design data to measure the entry point, apex point, and angular deviation.

**Results:**

The accuracy of group C was 1.36 ± 0.51 mm for entry point, 1.30 ± 0.59 mm for apex point, and 3.20 ± 0.74° for angular deviation. The accuracy of group M was 1.06 ± 0.31 mm for the entry point, 1.02 ± 0.30 mm for the apex point, and 2.91 ± 0.97° for angular deviation. Significant differences were observed in the entry and apex points between the two groups.

**Conclusions:**

The results indicate that group M exhibited better accuracy than group C, indicating that the stability of the thermoplastic clip is important for ensuring the accuracy of the dynamic navigation system. No previous studies have verified the accuracy of this system using the mouthpiece method, and additional data is required to confirm its accuracy for dental implant placement. The mouthpiece method improves the accuracy of implant placement and provides a safer implant treatment than the conventional method.

**Trial registration:**

University hospital Medical Information Network Clinical Trials Registry (UMIN-CTR), Registration Number: UMIN000051949, URL: https://center6.umin.ac.jp/cgi-open-bin/ctr_e/ctr_view_his.cgi on August 21, 2023.

## Background

The application of digital technology in dentistry is rapidly advancing. Intraoral scanners (IOS) and computer-aided design/computer-aided manufacturing (CAD/CAM) have reduced the treatment time and improved the accuracy of prosthetics [1–3]. Recently, the concepts of top-down treatment and static and dynamic navigation are widely employed to improve the accuracy of implant placement [4, 5]. Derksen et al. [6] reported a good static guide accuracy of 0.75 ± 0.34 mm for the entry point, 1.06 ± 0.44 mm for the apex point, and 2.72 ± 1.42° for angular deviation for 145 implants. However, static navigation has disadvantages such as overheating during drilling and a limited aperture [7, 8], whereas dynamic navigation enables implant placement without these risks. Navigation systems were first introduced to the market in 2001, with their use reportedly beginning in neurosurgery [9]; currently, their application has expanded to dental implant treatments. Wang et al. [10] compared freehand, static guide, and dynamic navigation on a model and reported that the accuracy of dynamic navigation was 1.09 ± 0.41 mm for the entry point, 1.55 ± 0.56 mm for the apex point, and 3.37 ± 1.56° for angular deviation, which was similar to that of the static guide and better than that of the freehand. X-Guide®, a dynamic navigation system, was launched in 2020 in Japan. X-Guide® provides drilling support using two cameras in a ramp that reads handpiece and patient trackers connected to an X-Clip—a small thermoplastic device with three radiopaque fiducial markers—fixed to the patient’s teeth to accurately triangulate the position of the handpiece in real time. The most important factor that ensures the implantation accuracy of X-Guide® is the stability of the X-Clip fixed to the tooth. However, the patient’s remaining teeth, as well as crown height and width diameters, vary [11]. Thus, if the X-Clip was fixed not to the tooth but to a mouthpiece, the implant treatment could be performed more safely and with better placement accuracy.

Therefore, this study aims to verify whether a difference exists in the accuracy between two groups: one in which the X-Clip is fixed to the tooth in a conventional manner, and the other in which the X-Clip is fixed to a mouthpiece attached to the tooth.

## Methods

Thirty-eight partially edentulous patients (17 males and 21 females) requiring implant treatment with a mean age of 55.3 years and 50 implants were selected. Both group C (conventional method) and group M (mouthpiece method) consisted of 19 patients and 25 implants. Patients undergoing X-Guide®-assisted surgery were fully informed about the conventional and mouthpiece methods and asked to choose one or the other; sampling was conducted until each group was made up of 25 patients. In accordance with the instructions stipulated by the university’s ethics committee, the procedure type selected by the patient was applied, instead of applying a random procedure type. Patients were required to be at least 20 years old, have no more than free-end or intermediate missing with three or less missing, and have no bone grafts. The implant placement sites were restricted to premolar and molar areas. All implant surgeries were performed after post-extraction healing had concluded at the site.

Intraoral data for all patients were obtained by digital impression in the IOS (Trios®3, 3Shape, Copenhagen, Denmark), and a digital wax-up of the defects was performed using CAD (Exocad®, Exocad, Berlin, Germany).

**Fig. 1:**
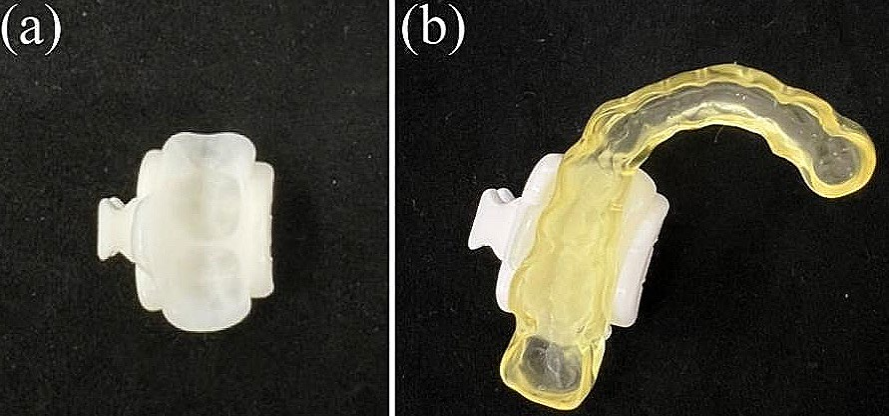
X-Clip used in (**a**) the conventional method and (**b**) mouthpiece method

In addition, cone-beam computed tomography (CBCT; 3DX, Morita, Tokyo, Japan) was used to capture intraoral images with the X-Clip fixed to the teeth or to a mouthpiece attached to the teeth, and Digital Imaging and Communications in Medicine (DICOM) data were obtained for each image. During imaging, both groups chewed dental cotton rolls to stabilize the X-Clips and to avoid the overlapping of the upper and lower dentition. These data for each patient were superimposed using DTX Studio™ (Nobel Biocare AG, Kloten, Switzerland), a treatment planning support program for dental implants, to determine the implant fixture selection and placement position. The preoperative implant design data were then exported and imported into X-Guide® (X-Nav technologies, Lansdale, PA, USA), a dynamic navigation system. X-Clips were softened by immersion in hot water at 60–71 °C for 8 min according to the manufacturer’s recommendations; if the softening was not sufficient, the temperature was adjusted and immersion was performed again. After confirming that the resin of the X-Clip was completely clear, the X-Clip was immersed in hot water at 40 °C for 1 min; then, it was pressure-applied to three molar teeth, either natural teeth or teeth with prosthetic treatment, in group C. In group M, the X-Clip was pressure-applied to the mouthpiece; then, the resin was cured in cold water (Fig. 1). The Kanagawa Dental University Ethics Committee approved this study (approval number: 905). Written informed consent was obtained from all patients.

### Mouthpiece fabrication

The form of the mouthpiece was determined using CAD from the patients’ intraoral data acquired by IOS, and a 12-millimeter-thick mouthpiece was fabricated using a 3D printer (Phrozen Sonic Mighty 4 K, Denken-Highdental Co., Ltd., Kyoto, Japan) and DH Print Sprint & Guide (Denken-Highdental Co., Ltd., Kyoto, Japan). For patients with missing intermediate teeth, the mouthpiece covered the more residual dentition, whereas for patients with free end defects, the mouthpiece covered the entire residual dentition. The length of the mouthpiece extended to the cervix dentis, and the spacer was set at 0.05 mm.

### X-guide® preparation

First, a hand tracker was attached to the handpiece, calibration disks were then attached to the handpiece, and a camera was used for reading and calibration. In addition, the patient tracker was fitted with an X-Clip, which was read into the camera for calibration. Finally, a drill was mounted on the handpiece, and the drill length was measured by enabling the tip to contact the Go plate perpendicularly and ensuring that the camera reads it. Subsequently, the drill tip was made to touch the three reference points on the X-Clip to confirm that the measurement was $\le \,0.2\,mm$, and the X-Guide preparation was completed.

### Surgical procedure

**Fig. 2:**
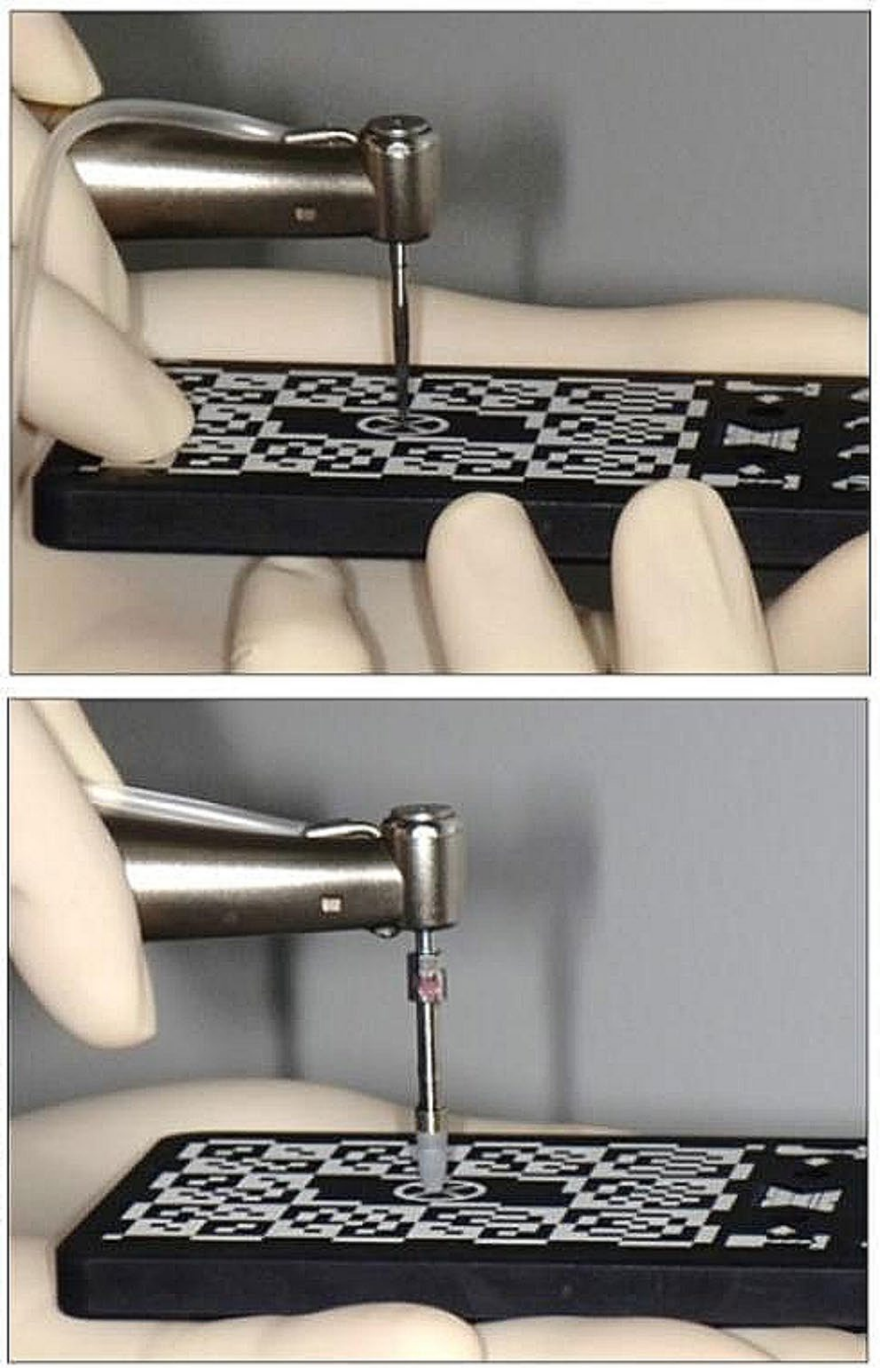
Measurement of drill and implant lengths. All drill and implant lengths were measured

All patients underwent infiltration anesthesia (Lidocaine/Adrenaline bitartrate®, Showa Yakuhin Kako Co., Ltd., Tokyo, Japan) and avulsion after gingival incision. Next, an X-Clip or a mouthpiece fixed with an X-Clip was placed on the tooth. Subsequent drilling was performed according to the manufacturer’s protocol for each process, with the handpiece and X-clip tracker recorded using two cameras and projected on a screen in real time to facilitate drilling. Before drilling, the drill length was always measured by enabling the drill tip to perpendicularly touch the Go plate. Similarly, before implant placement, the implant tip was made to vertically touch the Go plate; then, the length of the implant fixture was measured. Placement of the implant was performed using the X-Guide® software (Fig. 2). After placement of the implant fixture, the X-Guide® screen was used to confirm that the implant was correctly placed according to the preoperative implant design data. The implant system also used implant fixtures selectable in DTX Studio™. All surgeries were performed by one implant specialist.

### Accuracy measurement methods

**Fig. 3:**
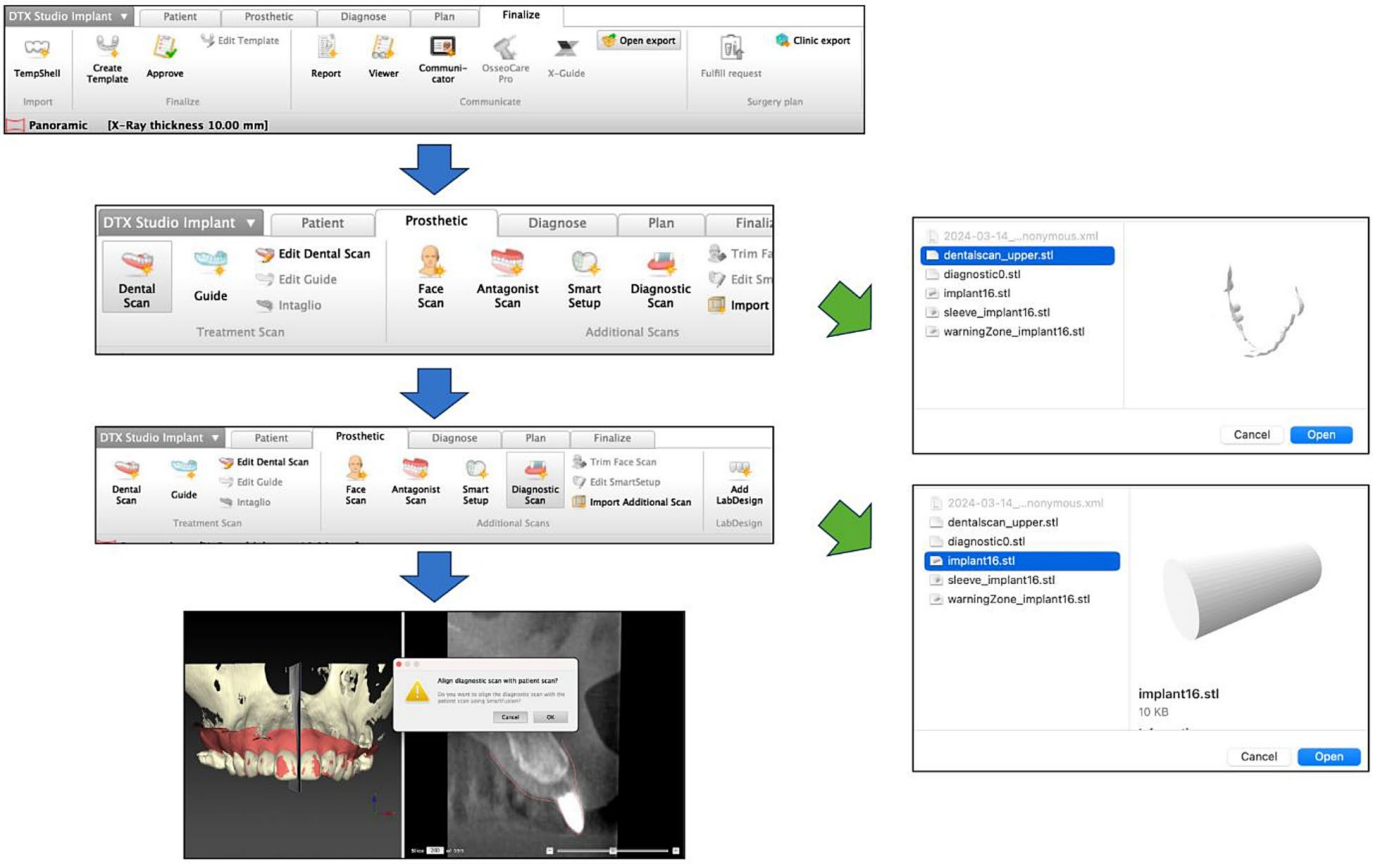
Methodology for superimposing preoperative implant design data and DICOM data after implant placement

The preoperative implant design data are exported as STL data by selecting “Open export” from “Finalize” in DTX Studio™. The DICOM data obtained from the post-implantation CBCT imaging were then imported into DTX Studio™, and the preoperative intraoral STL data “dentalscan” were selected from “Dental Scan” in the “Prosthetic” menu for superimposition. In this process, the postoperative DICOM data and preoperative implant design data were manually superimposed, using the remaining teeth as a reference. Then, “Diagnostic Scan” was selected and the STL data of “implant” were chosen. Then, “Align diagnostic scan with patient scan” appeared on the screen. Finally, “Cancel” was selected, and the process was complete. This allowed the preoperative implant design data to be superimposed onto the postoperative implant data (Fig. 3).

**Fig. 4:**
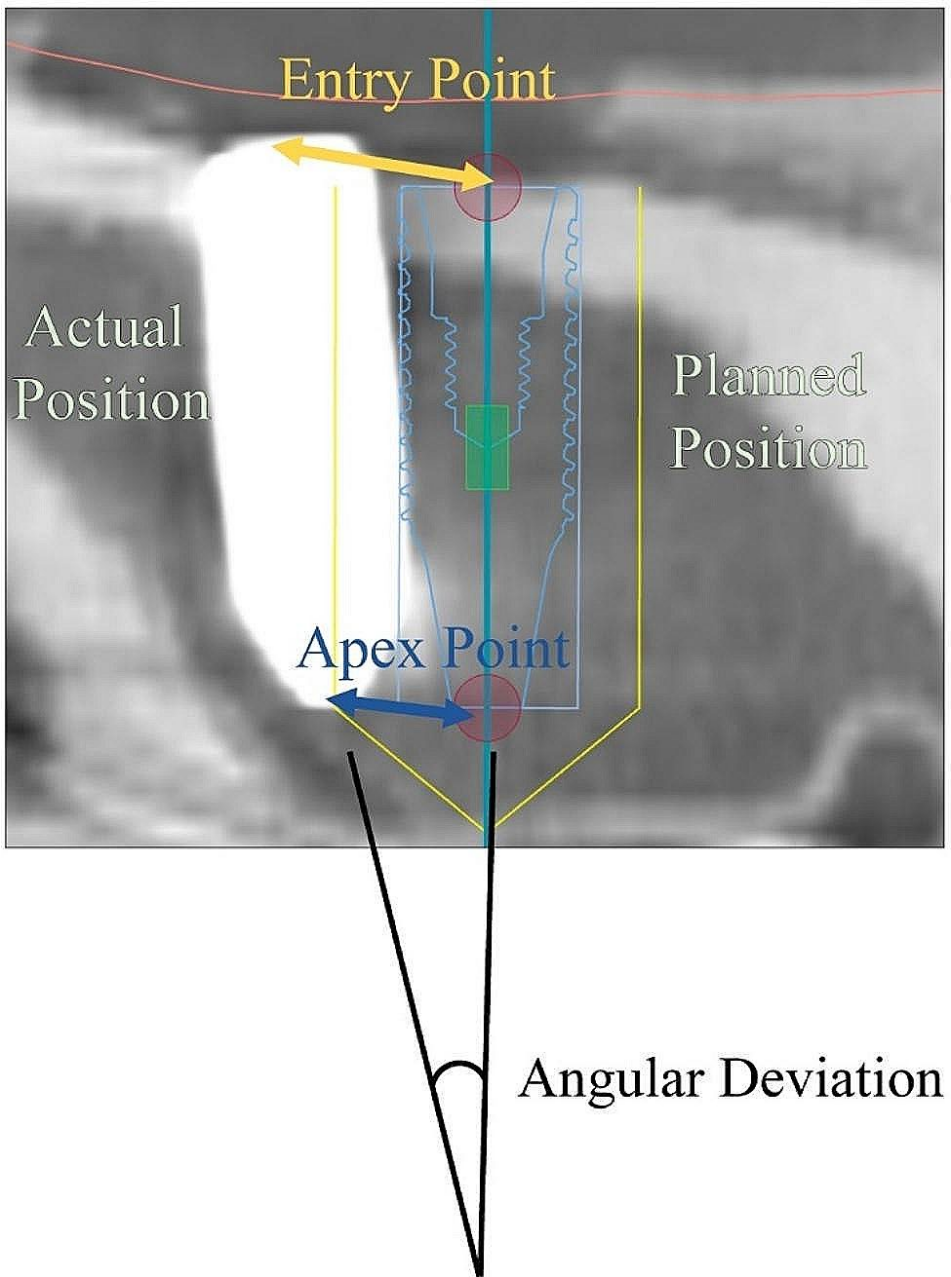
Accuracy and angle measurement method

After superimposition, the entry point, apex point, and angular deviation were measured at two locations, parallel and perpendicular to the dentition, for accuracy and angle, and the average value was used as the result (Fig. 4).

### Statistical analysis

G-Power (version 3.1.9.2) was used to perform one-way analysis of variance. The sample size required to obtain 80% of the effect size of 0.8 at α = 0.05 was calculated. A student’s t-test was used to compare groups C and M using a bell curve from Microsoft Excel (Social Survey Research Information Corporation, Tokyo, Japan). *P*-values < 0.05 were considered significant.

## Results

The accuracy was 1.36 ± 0.51 mm for the entry point, 1.30 ± 0.59 mm for the apex point, and 3.20 ± 0.74° for angular deviation for group C, and that for group M was 1.06 ± 0.31 mm for the entry point, 1.02 ± 0.30 mm for the apex point, and 2.91 ± 0.97° for angular deviation. A large difference existed between the entry and apex points in both groups (*P* < 0.05) (Table 1).

**Table 1:** Results of this study. *P*-values < 0.05 were considered significant

Treatment	Patients	Implants	Entry point (mm)	Apex point (mm)	Angular deviation (°)
Group C	19	25	1.36 ± 0.51	1.30 ± 0.59	3.20 ± 0.74
Group M	19	25	1.06 ± 0.31	1.02 ± 0.30	2.91 ± 0.97
*P*-value	-	-	0.03	0.04	0.23

## Discussion

In this study, we assumed that group M would yield more stable X clips during surgery; therefore, we distinguished between group M and group C and compared their accuracy. Although a few reports are available on accuracy using X-Guide®, Emery et al. [12] verified the accuracy of X-Guide® on dental models and reported an overall error of 0.35 ± 0.16 mm for the entry point, 0.31 ± 0.16 mm for the apex point, and 1.0 ± 0.4° for angular deviation in three dimensions. Block et al. [13] also validated the accuracy of X-Guide® in clinical practice with 478 patients and 714 implants. They reported that, when using the X-Guide® software for implant placement, the entry point was 1.16 ± 0.59 mm, apex point was 1.29 ± 0.65 mm, and angular deviation was 2.97 ± 2.09°. The accuracy in clinical practice tended to be lower than that in model studies. Kaewsiri et al. [14] compared the accuracies of dynamic navigation (IRIS-100) and static guide in 60 patients with single defects. The accuracy of dynamic navigation was 1.05 ± 0.44 mm for the entry point, 1.29 ± 0.50 mm for the apex point, and 3.06 ± 1.37° for angular deviation, and that for the static guide group was 0.97 ± 0.44 mm for the entry point, 1.28 ± 0.46 mm for the apex point, and 2.84 ± 1.71° for angular deviation, indicating only small differences in accuracy between the two. Yimarj et al. [15] compared the placement position and parallelism accuracy in 60 patients who required two consecutive implants using a static guide and dynamic navigation (IRIS-100). The accuracy of dynamic navigation was 1.24 ± 0.39 mm for the entry point, 1.58 ± 0.56 mm for the apex point, and 3.78 ± 1.84° for the angular deviation, while that of the static guide was 1.04 ± 0.67 mm for the entry point, 1.54 ± 0.79 mm for the apex point, and 4.08 ± 1.69° for angular deviation, indicating negligible differences. However, the angular difference between the two implants was 3.55 ± 2.29° for dynamic navigation and 4.32 ± 2.44° for static guide, indicating that dynamic navigation was better at maintaining parallelism. These reports suggest that the accuracy of the two guides is almost the same, but that parallelism of the static guide was reduced owing to the distortion of the surgical guide during drilling [16]. As shown in these reports, the advantage of dynamic navigation is that there is no aperture limitation, and parallelism can be maintained. Therefore, we considered that the angular deviation was not affected and that no significant difference existed between the two groups. While these reports used some dynamic navigation, a systematic review by Wei et al. [17] reported no difference between Navident (ClaroNav, Toronto, Canada), X-Guide®, Aq Navi (Taiwan Implant Technology Co., Ltd., Kaohsiung, Taiwan), ImplaNav™ (BresMedical Pty Ltd., Ingleburn, Australia), IRIS (EPED Inc., Kaohsiung, Taiwan), and these five systems. In all 10 included studies, preoperative DICOM data, preoperative implantation planning position, and postoperative DICOM data were measured for accuracy using 3D data-editing software. However, the process of superimposing these three sets of data is complex, and artifacts can adversely affect the accuracy [18]. In this study, the mouthpiece improved the accessibility of group M, which we postulate is the reason for the good accuracy. However, to the best of our knowledge, this is the first study to report on preoperative intraoral and implant design data exported as STL data and superimposed on postoperative DICOM data for measurement. When exporting preoperative implant design data as a STL dataset, the implant design data is combined and exported together with the tooth and mucosa data of the mouth. The automatic superimposition of the STL data with the postoperative DICOM data in DTX Studio^™^ is not expected to affect accuracy. Accuracy would only be impacted if an error occurred when superimposing the preoperative DICOM and STL data. However, this error would cause the implant surgery to fail, in which case navigation surgery should be avoided and freehand surgery should be performed. Although measuring accuracy without applying 3D data editing software simplifies postoperative feedback, more reliable measurement methods need to be validated in the future.

When using dynamic navigation, preoperative DICOM, intraoral STL, and wax-up data were superimposed to determine the placement position [10, 12, 13]. Good data have been reported for these superimpositions using treatment planning assistance programs for dental implants [19, 20]. We consider that the factors that affect the accuracy of X-Guide® are body motion during CT imaging and the stability of the X-Clip during surgery. CT and CBCT have been used in dentistry for many years, and their accuracy has been verified using various methods, demonstrating their practicality in clinical practice [21, 22]. Kyme et al. [23] reported that patient motion during CT imaging appears as artifacts during data reconstruction, degrading image quality and impairing accurate image interpretation and quantification. Kaasalainen et al. [24] reported that the acceptable range of body motion is 500 μm when all relevant factors are considered during CBCT imaging, but it also depends on the duration of irradiation, device used, and age, and it varies among individuals. Considering these factors, it is conceivable that errors could have occurred in both groups T and M. However, because the imaging conditions were the same for all patients, we believe that the stability of the X-Clip during implant surgery is a factor that influenced the accuracy the most. Regarding crown size, Song et al. [25] reported crown width and height diameters of 7.47 ± 0.53 mm and 8.11 ± 1.02 mm, respectively, for the maxillary first premolars, 7.03 ± 0.43 mm and 6.94 ± 1.02 mm, respectively, for the second premolars, 10.62 ± 0.59 mm and 6.68 ± 0.7 mm, respectively, for the first molars, and 7.19 ± 0.63 mm and 6.57 ± 0.7 mm, respectively, for the second molars. Hiltunen et al. [26] reported that 8.5% of the 282,143 patients, aged 60 years and older, surveyed between 2007 and 2017 were treated with prosthetic therapy. Cenzato et al. [27] reported that plexus malocclusion is the most common form of malocclusion; however, its proportion is higher in the anterior teeth and almost absent in molars. Therefore, the oral cavities of each patient differed. Whether the oral condition of the X-Clip is the most stable is unknown. Because group M could suppress the movement of the X-Clip during implant surgery without being affected by the patient’s oral environment, we conclude that the accuracy of group M was higher than that of group C. No studies have verified the accuracy of X-Guides using the mouthpiece method. Therefore, additional data must be accumulated to verify the accuracy.

## Conclusions

In this study, the accuracies of conventional and mouthpiece methods were compared. Preoperative implant design data were discharged as STL data and superimposed on postoperative CT data to measure accuracy, suggesting that implant surgery was performed more safely in group M, independent of the patient’s oral environment. These findings suggest that further studies should be performed to verify the accuracy of the mouthpiece method for dental implant placement using X-Guide®, which may lead to improved safety and accuracy during dental implant surgery in clinical practice.

## Data Availability

The datasets used and analyzed during the current study are available from the corresponding author on reasonable request.

## Abbreviations

IOS: Intraoral scanners
CAD/CAM: Computer-aided design/computer-aided manufacturing
CBCT: Cone-beam computed tomography
DICOM: Digital Imaging and Communications in Medicine
